# Novel peptides derived from neuropeptide Y prevent chemotherapy-induced bone marrow damage by regulating hematopoietic stem cell microenvironment

**DOI:** 10.1080/19768354.2018.1517826

**Published:** 2018-09-05

**Authors:** Min Hee Park, Bosung Baek, Hee Kyung Jin, Jae-sung Bae

**Affiliations:** a Stem Cell Neuroplasticity Research Group, Kyungpook National University, Daegu, South Korea; b Department of Physiology, Cell and Matrix Research Institute, School of Medicine, Kyungpook National University, Daegu, South Korea; c Department of Biomedical Science, BK21 Plus KNU Biomedical Convergence Program, Kyungpook National University, Daegu, South Korea; d Department of Laboratory Animal Medicine, College of Veterinary Medicine, Kyungpook National University, Daegu, South Korea

**Keywords:** Bone marrow damage, bone marrow microenvironment cells, Chemotherapy, hematopoietic stem cell, neuropeptide Y-derived recombinant peptides

## Abstract

Chemotherapy-induced bone marrow damage is accompanied by acute nerve injury in the bone marrow (BM), resulting in sensory and autonomic neuropathy. Cisplatin, a popular chemotherapy drugs, induces the impairment of hematopoietic stem cells (HSCs) and bone marrow regeneration, leading to chronic bone marrow abnormalities. Previously, we reported the protective roles of neuropeptide Y (NPY) against cisplatin-induced bone marrow impairment. In this study, we identified novel peptides, generated from full-length NPY that rescued cisplatin-induced sensory neuropathy and HSC suppression by regulating cell survival in the BM microenvironment. One of these peptides, especially, showed a better protective property against these impairments compared to that seen in full-length NPY. Therefore, we suggest the NPY sequences most effective against the chemotherapy-induced bone marrow dysfunction that could be potentially useful as therapeutic agents for patients receiving chemotherapy.

## Introduction

Chemotherapy-induced hematopoietic dysfunction is one of the severe side effects seen in patients with cancer (Noach et al. 2000; Banfi et al. 2001; Kemp et al. 2010). The hematopoietic cells are constantly produced in the bone marrow (BM) by renewal, proliferation, and differentiation of the hematopoietic stem cells (HSCs) (Scadden 2006; Ding et al. 2012). Patients that have previously received chemotherapy, show dysfunction of HSCs and bone marrow regeneration, and delayed engraftment after bone marrow transplantation, leading to irreversible and chronic bone marrow failure (Mauch et al. 1995; Noach et al. 2000; Banfi et al. 2001; Kemp et al. 2010). Recently, chemotherapy-induced nerve injury in the BM was shown to be a critical lesion impairing hematopoietic regeneration. Cisplatin, which is one of the most widely used chemotherapeutic drugs, causes severe peripheral neuropathy by reducing expression of sympathetic nerve fibers in BM, consequently leading to functional defects in HSCs (Lucas et al. 2013). Therefore, nerve protection as a potential therapeutic application is on the rise for prevention of long-term bone marrow injury from chemotherapy.

Neuropeptide Y (NPY), which is consisted of 36-amino-acid, is an important neurotransmitter secreted from the brain or sympathetic nerves in the autonomic system and is involved in a variety of mechanisms related to metabolic physiology (Zukowska-Grojec 1995; Kalra and Kalra 2004). In a previous study, we reported a new role of NPY as a regulator of bone marrow microenvironment. Deficiency of NPY caused reduction in HSC survival, mobilization, bone marrow regeneration, as well as integrity of sympathetic nerve fibers in BM (Park, Jin et al. 2015; Park, Min et al. 2015, 2016). On the contrary, NPY treatment promoted neuroprotection and restored sensory neuropathy, HSC failure, and cell damage from chemotherapy in bone marrow microenvironment, suggesting the therapeutic potential of NPY against chemotherapy-induced bone marrow dysfunction (Park, Jin et al. 2015; Park, Min et al. 2015).

In this study, we revealed short functional peptides, containing the effective sequences of full-length NPY, capable of efficiently preventing chemotherapy-induced bone marrow dysfunction. Two such peptides were identified to be noticeably effective against chemotherapy-induced bone marrow dysfunction, out of which, one showed greater beneficial effects than the full-length NPY itself, suggesting its potential therapeutic application in patients treated with chemotherapy.

## Materials and methods

### Mice

Six- to eight-week-old male or female C57BL/6 mice were purchased from the Jackson Laboratory (Bar Harbor, ME). The animals were allocated to different experimental groups by block randomization method. To eliminate bias, investigators were blinded during data collection and analysis. The mice were housed with a 12-h day-night cycle and free access to tap water and food pellets. All mouse studies were approved by the Kyungpook National University Institutional Animal Care and Use Committee.

### Reagents treatment

Cisplatin (Enzo; 10 mg per kg of body weight, once per week) was used for chemotherapy, and the mice received i.p. injections of cisplatin for 7 weeks. To investigate the protective effect of NPY-derived peptides against cisplatin-induced bone marrow dysfunction, the full-length NPY (NPY(1–36), H-6375; Bachem, Bubendorf, Switzerland) was cleaved and recombined to give NPY (1-15), NPY (6-20), NPY (11-25), and NPY (21-36), all obtained from Peptron, Inc. (Daejeon, Korea). The mice received i.p. injections of each of the peptide daily during the 7-week cisplatin-treatment. An hour after the last injection, the BM and blood were collected and analyzed. For in vitro experiments, 10 nM of each peptide was applied to the macrophages cultured from BM of WT mice. Three days later, the cells were collected for RNA extraction, and CM collected for ELISA or PC12 cell differentiation. PC12 cells (Korean cell line bank, 21721, Seoul, Korea) cultured in DMEM supplemented with 10% FBS, 1% penicillin, and streptomycin (all from Gibco) were exposed to the CM derived from each peptide-treated macrophage to determine its influence on differentiation. Seven days later, PC12 cells with at least one or two neurite with a length equal to the cell body diameter was scored under an Olympus IX71 microscope.

### Flow cytometry

BM from the tibiae and femurs of each mouse were flushed. Red blood cells (RBCs) were lysed for 5 min at 4 °C in 0.15 M NH_4_Cl (STEMCELL Technologies, Vancouver, Canada), washed with PBS (Gibco), and counted using a hemocytometer. For HSC, MSC, or osteoblast detection, Lin^+^ cells were removed by magnetic depletion using biotinylated lineage-specific antibodies (CD5, CD45R, CD11b, Gr-1, and Ter-119), followed by depletion with MACs beads conjugated to monoclonal anti-biotin (Miltenyi Biotec, Gladbach, Germany). For staining of HSCs, Lin^−^ cells were stained with phycoerythrin (PE)-Cy7-conjugated antibodies to Sca1 (558162), APC-conjugated antibodies to c-Kit (553356), FITC-conjugated antibodies to CD48 (557484), and PE-conjugated antibodies to CD150 (561540), all from BD Biosciences. Cells were further stained with streptavidin-pacific blue (PB) (Invitrogen, S11222). Data were collected using AriaIII systems (BD Biosciences) and analyzed using FlowJo software (Tree Star, Ashland, OR).

### Immunofluorescence staining of bone marrow sections

Frozen bone marrow sections were prepared and immunostained according to a previously published method (Kawamoto 2003). Bone marrow sections were ﬁxed using dry ice/hexane. Sections were incubated first with a primary antibody, followed by incubation with secondary antibody conjugated with Alexa488 (Life Technologies, Carlsbad, CA). Immunoﬂuorescence data were obtained and analyzed using a laser scanning confocal microscope equipped with Fluoview SV1000 imaging software (Olympus FV1000, Japan). The antibodies used were as follows: Th (Millipore, Billerica, MA; AB152 or AB318, 1:250 dilution) and CD31 (BD Biosciences, 550300, 1:50 dilution). TUNEL assay was performed using the DeadEnd™Fluorometric TUNEL System (Promega Madison, WI) or *In Situ* Cell Detection Kit, TMR red (Roche, Basel, Switzerland) following the manufacturer’s instructions.

### Macrophage culture

The tibiae and femurs of adult mice were aseptically removed and dissected free of adhering tissues. The bones were cut off with scissors, and the marrow cavity was flushed with α-MEM (Gibco) by slowly injecting at one end of the bone using a sterile needle. The marrow cells were collected, washed with α-MEM, and red blood cells removed by the treatment of 0.15 M NH_4_Cl. After washing, the cells were cultured in α-MEM containing 10% FCS, 1% penicillin and streptomycin, and macrophage colony stimulating factor (M-CSF, R&D Systems, Minneapolis, MN; 100 ng/ml) at 5 × 10^6^ cells in a 10 cm suspension culture dish to which stromal and lymphoid cells cannot adhere. After 3 days, cells were washed vigorously twice with PBS to remove the non-adherent cells, harvested by pipetting with 0.02% EDTA in PBS, and seeded at 3 × 10^5^ cells in a 10-cm dish. After another 3 days, cells were obtained with approximately 10-fold increase in number compared to that during seeding. We used these cells as M-CSF-dependent BM macrophage cells, as described later (Takeshita et al. 2000).

### ELISA

TGF-β levels were assayed by using mouse TGF-β (R&D systems, Minneapolis, MN) according to the manufacturer’s instructions.

### Quantitation of sensory neuropathy by the heated-pad assay

To evaluate the effect of different treatments on the sensory response, we performed the hot-plate test as previously described (Aloe et al. 2000). We used a heating apparatus (Panlab/Harvard Apparatus, Barcelona, Spain) maintained at 50 °C. Mice were individually placed on the heated surface, and the time of the first episode of nociception (jumping or paw licking) was noted. The cutoff time was 60 s. Each test was repeated three times at an interval of 15 min, and the median values were analyzed. Between any two measurements, the heated surface was thoroughly cleaned with detergent and ethanol, and the temperature was allowed to stabilize at 50 °C.

### Quantitative real-time PCR

RNA was extracted from BM using the RNeasy Lipid Tissue Mini kit (Qiagen, Hilden, Germany) according to the manufacturer’s instructions. cDNA was synthesized from 5 μg of total RNA using a commercially available kit from Clontech (Mountain View, CA, USA). Quantitative real-time PCR was performed using a Corbett Research RG-6000 real-time PCR instrument. The following mouse primers were used: TGF-β (forward, 5′-CACCCACTTTTGGATCTCAG-3′; reverse, 5′-CCCAAGGAAAGGTAGGTGAT-3′) and GAPDH (forward, 5′-TGGCAAAGTGGAGATTGTTGCC-3′; reverse, 5′- AAGATGGTGATGGGCTTCCCG-3’).

### Statistical analysis

Comparisons between more than two groups were performed using one-way analysis of variance (ANOVA), followed by Tukey’s HSD test. All statistical analyses were performed using SPSS statistical software. *P < 0.05, **P < 0.01, ***P < 0.001 were the markers of statistical significance.

## Results

### NPY (1–15) and NPY (6–20) generated from full-length NPY prevent cisplatin-induced HSC reduction in BM

**Figure 1.**
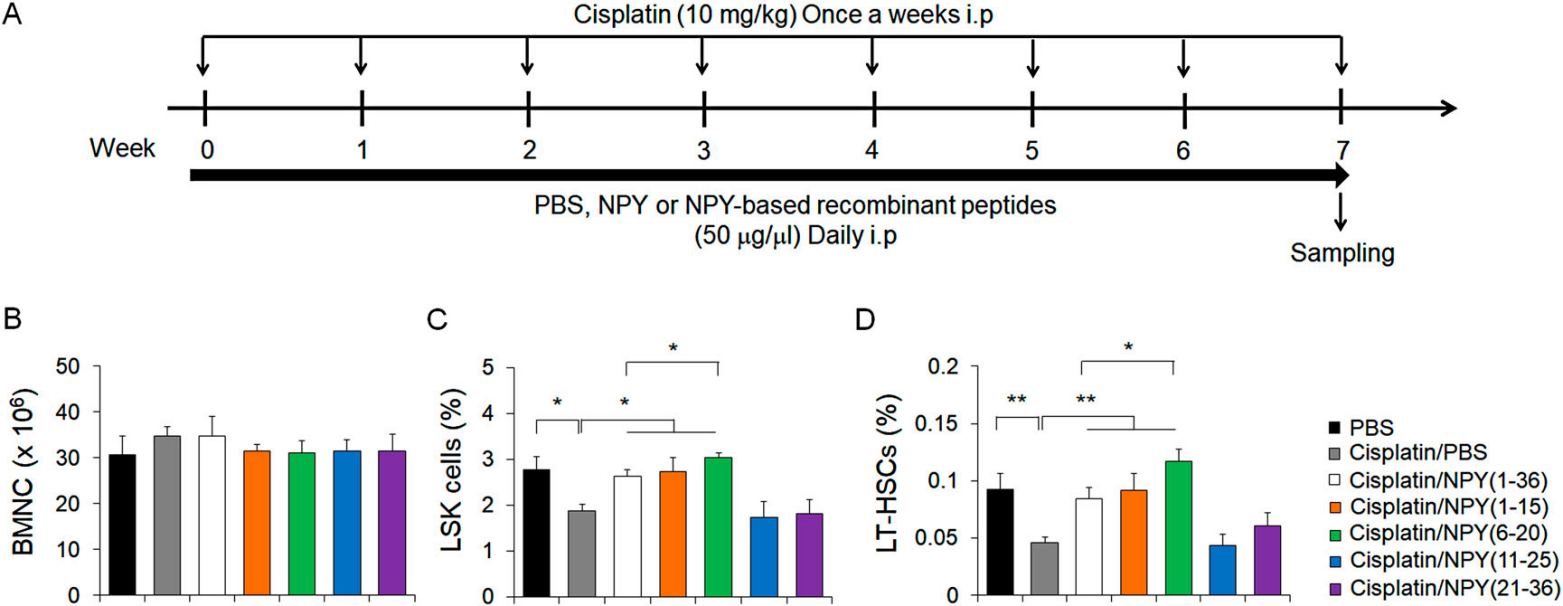
NPY (1–15) and NPY (6–20) mitigate the cisplatin-induced reduction in HSC abundance in BM. (A) Experimental design to investigate the effect of NPY-derived peptides in cisplatin-induced bone marrow dysfunction. Mice were intraperitoneally treated with PBS (daily), 10 mg/kg cisplatin (once a week), or 10 mg/kg cisplatin (once a week) plus 50 μg/kg NPY or NPY- derived peptides (daily). (B-D) Number of (B) BMNCs and percentage of (C) LSK cells or (D) LT**-**HSCs in BM of cisplatin with PBS-, NPY (1–36)-, NPY (1–15)-, NPY (6–20)-, NPY (11–25)- or NPY (21–36)- treated mice (*n* = 7 per group). *P < 0.05, **P < 0.01. All error bars indicate the standard errors of the mean (S.E.M.).

To reveal the stretches of NPY sequence responsible for the improvement of chemotherapy-induced bone marrow dysfunction, we selected four different fragments of the full-length NPY (1–36): NPY (1–15), NPY (6–20), NPY (11–25), and NPY (21–36). Wild-type (WT) mice treated with seven cycles of cisplatin were administered either full-length NPY (1–36) or any of the four different peptides during cisplatin chemotherapy (Figure 1A). No significant difference in the number of BMNCs was observed across the groups (Figure 1B). Of note, cisplatin-induced reduction of Lin^−^ Sca-1^+^ c-Kit^+^ (LSK) cells and long-term HSCs (LT-HSC; LSK CD48^−^ CD150^+^) were recovered in NPY (1–15)- and NPY (6–20)-treated mice (Figure 1C and D). Particularly, the mice treated with NPY (6–20) showed greater restoration of impaired HSC than those treated with NPY (1–36). These results indicate that NPY (6–20) is potent in preventing chemotherapy-induced HSC damage more effectively.

### NPY (1–15) and NPY (6–20) improve the cisplatin-triggered sensory neuropathy and cell death in bone marrow microenvironment

**Figure 2.**
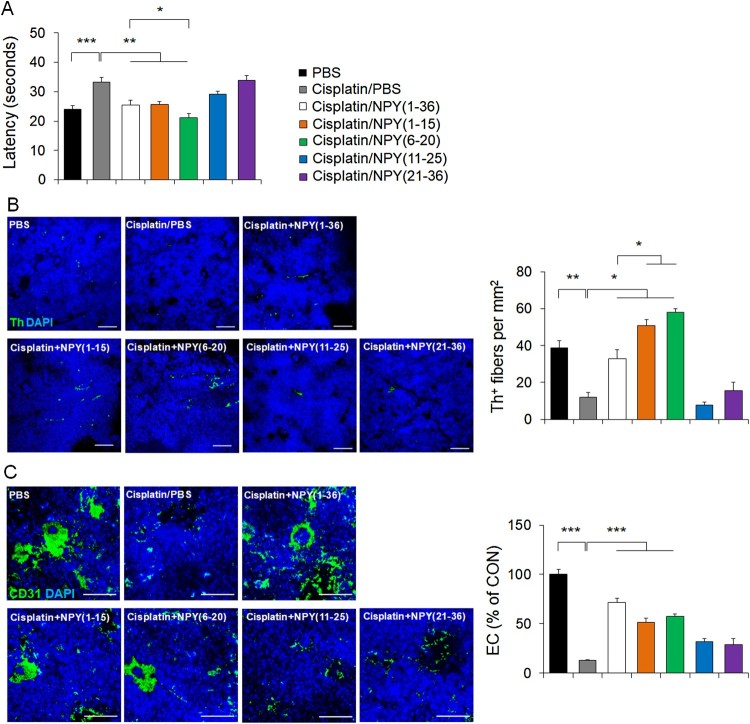
NPY (1–15) and NPY (6–20) improve cisplatin-induced sensory neuropathy and death of SNS fibers and ECs in the BM. (A) Quantification of sensory neuropathy (2 weeks after the last cisplatin injection) in each group (*n* = 10 per group). Latency indicates the time before the mouse showed signs of nociception (jumping or paw licking). (B) Left, representative immunofluorescence images to detect the presence of Th^+^ fibers. Scale bar, 50 μm. Right, quantification of Th^+^ fibers in the BM of each group (*n* = 4–5 per group). (C) Left, representative immunofluorescence images to detect the presence of CD31^+^ ECs. Scale bar, 20 μm. Right, quantification of CD31^+^ ECs in the BM of each group (*n* = 4–5 per group). *P < 0.05, **P < 0.01, ***P < 0.001. All error bars indicate S.E.M.

Previous studies demonstrated that chemotherapy-induced nerve injury in BM leads to sensory neuropathy (Aloe et al. 2000; Cavaletti and Marmiroli 2010; Lucas et al. 2013). Moreover, it causes HSC dysfunction by decreasing the bone marrow cells related to HSC survival such as endothelial cells (ECs) (Butler et al. 2010; Lucas et al. 2013). Recently, our group also reported that NPY maintains HSC function by protecting sympathetic nervous system (SNS) fiber and EC survival in BM (Park, Jin et al. 2015; Park, Min et al. 2015). To confirm that NPY (1–15) and NPY (6–20) also possess protective properties against cisplatin-induced sensory neuropathy, experimental mice were individually placed on a heated apparatus maintained at 50°C, and the time of the first episode of nociception (jumping or paw licking) was recorded. Cisplatin-induced sensory neuropathy was found to be improved by NPY (1–15) and NPY (6–20) treatment (Figure 2A). The cisplatin-triggered decrease of the SNS fibers, which were stained with an antibody against the catecholaminergic enzyme, tyrosine hydroxylase (Th), was prevented in the NPY (1–15)- and NPY (6–20)-treated mice compared to that in mice treated with cisplatin alone (Figure 2B). Interestingly, NPY (6–20) showed a better neuroprotective effect against cisplatin than that seen with NPY (1–36). The reduction of ECs in BM was also prevented in both NPY (1–15)- and NPY (6–20)- treated mice (Figure 2C).

**Figure 3.**
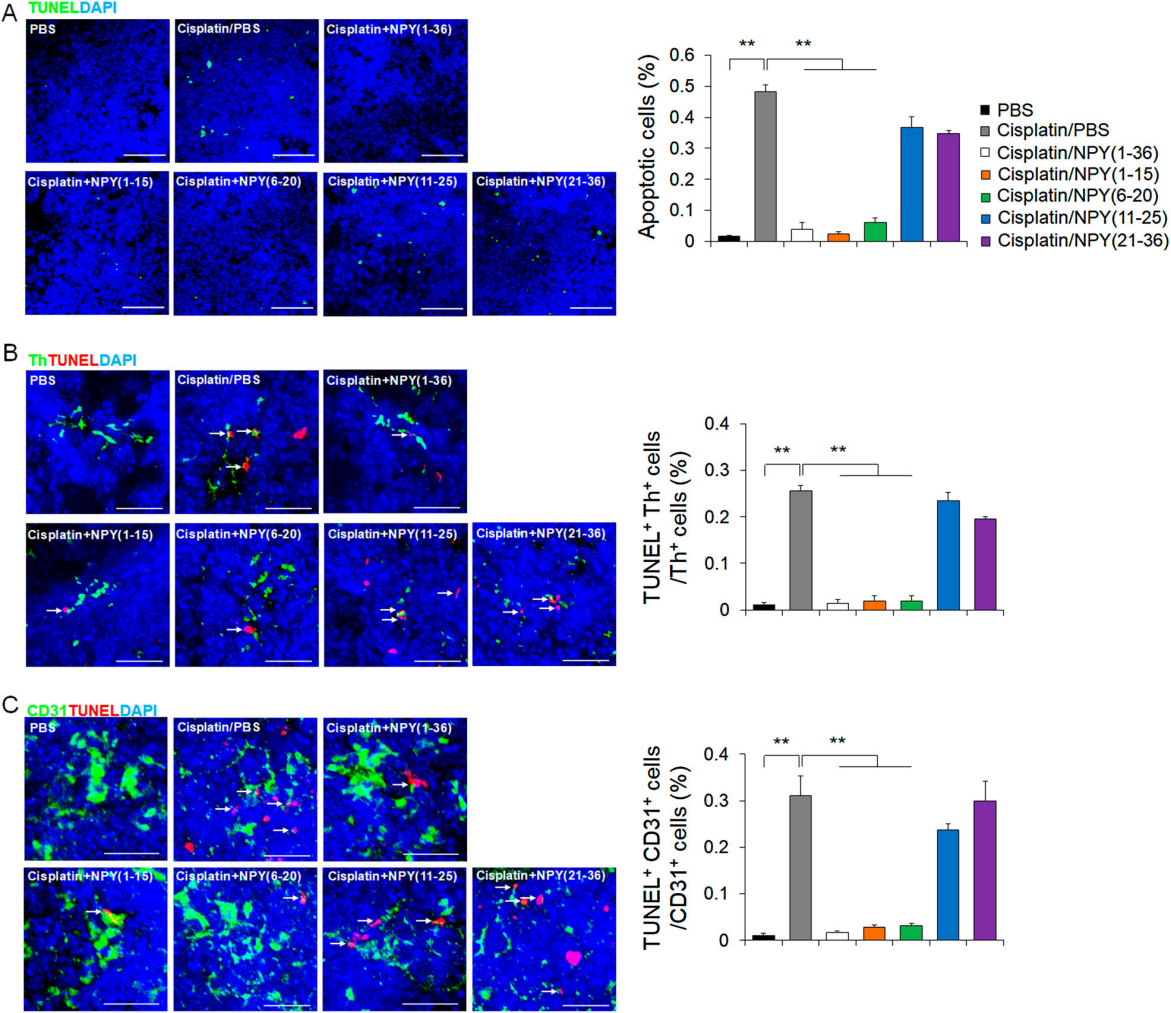
NPY (1–15) and NPY (6–20) protect cisplatin-induced apoptosis of SNS fibers and ECs in the BM. (A) Left, representative immunofluorescence images to detect the apoptotic cells by TUNEL staining. Scale bar, 20 μm. Right, quantification of apoptotic cells in BM of each group (*n* = 4 per group). (B) Representative immunofluorescence images of BM showing apoptosis by TUNEL stain (red) in sympathetic neuron (Th, green, Scale bar, 20 μm, arrow: apoptotic Th^+^ fibers). Right, quantification of apoptotic Th^+^ fibers in BM of each group (*n* = 4 per group). (C) Representative immunofluorescence images of BM showing apoptosis by TUNEL stain (red) in CD31^+^ ECs (green, Scale bar, 20 μm, arrow; apoptotic CD31^+^ ECs). Right, quantification of apoptotic CD31^+^ ECs in BM of each group (*n* = 4 per group). **P < 0.01. All error bars indicate S.E.M.

Cisplatin induces cytotoxicity directly through cell-death signaling, and NPY is involved in the cell-death mechanism (Santos-Carvalho et al. 2013; Park, Jin et al. 2015; Park, Min et al. 2015). We observed increased apoptosis levels in the BM of cisplatin-treated mice, and most of it overlapped with that seen for Th^+^ cells or CD31^+^ ECs. However, in the cisplatin-plus-NPY (1–15)- or -NPY (6–20)-treated mice, significant reduction in the apoptotic Th^+^ cells or CD31^+^ ECs were seen, similar to that in case of NPY (1–36) (Figure 3A-C). Taken together, these results suggest that NPY (1–15) and NPY (6–20) can prevent sensory neuropathy and cell damage in bone marrow microenvironment.

### NPY (1–15) and NPY (6–20) enhance neuroprotection by promoting TGF-β secretion from macrophages

**Figure 4.**
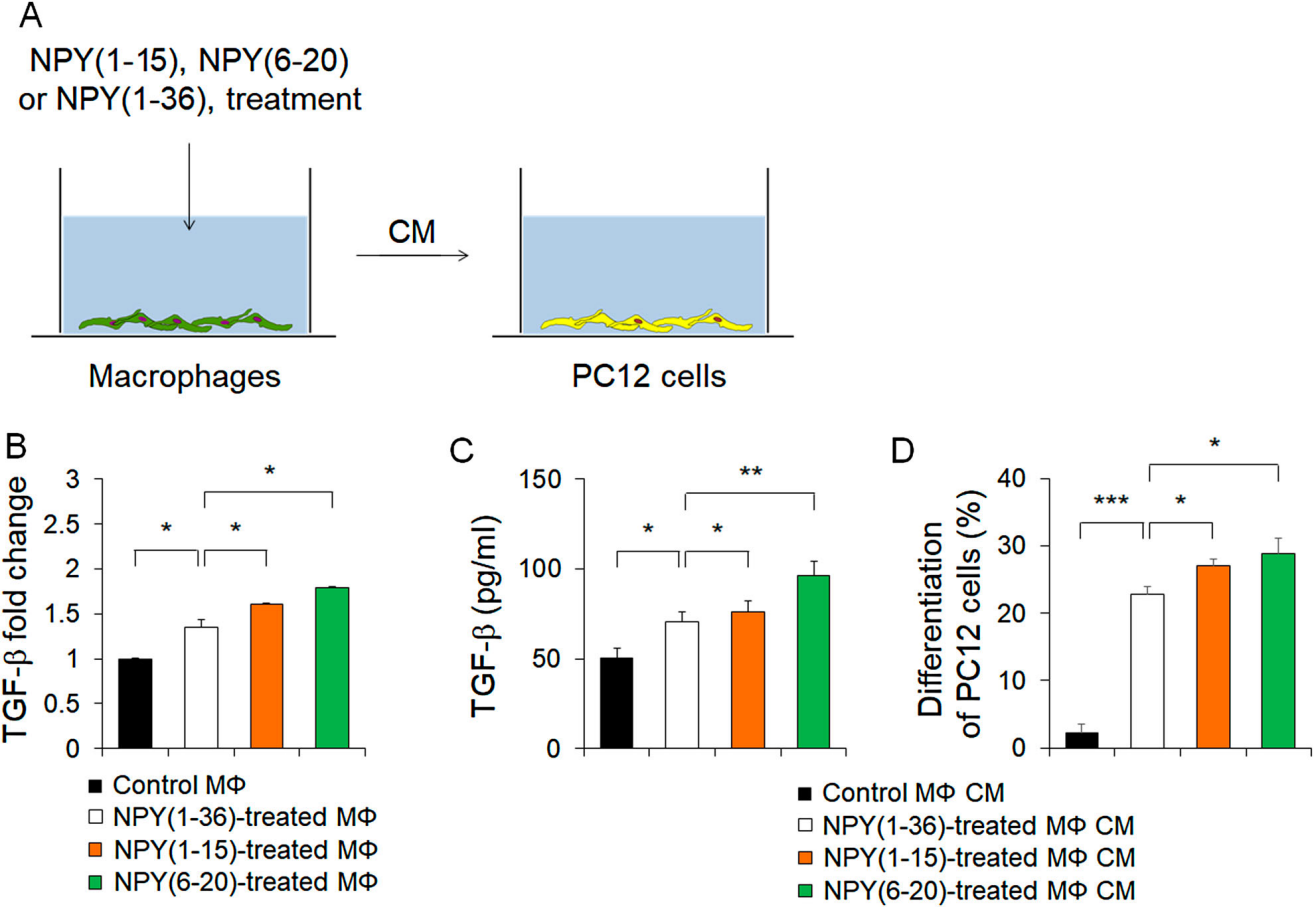
NPY (1–15) and NPY (6–20) up-regulate TGF-β secretion from macrophages and promote neural differentiation. (A) Illustration for the investigation of neural differentiation using CM derived from NPY (1–15)- and NPY (6–20)-treated macrophages. (B) mRNA levels of TGF-β in NPY (1–36)-, NPY (1–15)-, and NPY (6–20)-treated macrophages (*n* = 4 per group). (C) Protein levels of TGF-β in CM derived from NPY (1–36)-, NPY (1–15)-, and NPY (6–20)-treated macrophages (*n* = 4 per group). (D) Quantification of the percentage of PC12 cells differentiated into neurons after incubation with CM derived from NPY (1–36)-, NPY (1–15)-, and NPY (6–20)-treated macrophages (*n* = 5 per group). *P < 0.05, **P < 0.01, ***P < 0.001. All error bars indicate S.E.M. All expression levels are relative to that of Gapdh mRNA.

TGF-β is known to regulate of neuronal survival, differentiation, and repair processes in the nervous system (Krieglstein et al. 2002; Xiao et al. 2008; Knöferle et al. 2010). A previous study reported that NPY regulated TGF-β production via Y1 receptor (Zhou et al. 2008), and we recently demonstrated that TGF-β released by NPY-mediated Y1 receptor stimulation in macrophages promoted neuroprotection from cisplatin-induced injury (Park, Jin et al. 2015; Park, Min et al. 2015). Based on the previous studies by our and other groups, we confirmed the possibility of neuroprotection by NPY (1–15) and NPY (6–20). We first cultured macrophages from the BM of WT mice and treated these macrophages with NPY (1–15), NPY (6–20) or NPY (1–36). Next, conditioned medium (CM) harvested from each group was applied to PC12 cells to determine the influence on neural differentiation (Figure 4A). We found increased TGF-β mRNA levels in NPY (1–15)- or NPY (6–20)-treated macrophages; elevated TGF-β secretion was seen in the CM derived from NPY (1–15)- or NPY (6–20)-treated macrophages (Figure 4B and C). Moreover, CM derived from NPY (1–15)- or NPY (6–20)-treated macrophages was better able to induce differentiation of PC12 cells towards neurons, compared to that from control macrophages (Figure 4D). Therefore, these findings suggested that NPY (1–15) and NPY (6–20) induce TGF-β secretion from macrophages, and protect cisplatin-induced cell damage in BM microenvironment. Since NPY (6–20) was more effective in neuroprotection against the cisplatin-induced injury than full-length NPY in BM, it can be considered to be the most effective NPY sequence for rescuing the chemotherapy-induced bone marrow dysfunction.

## Discussion

Bone marrow microenvironment consists of functional molecules and cells that regulate HSC retention, survival, self-renewal, and differentiation (Scadden 2006; Ding et al. 2012). Chronic bone marrow damage by chemotherapy accompanies reduced recovery of the bone marrow microenvironment cells, hence impairing hematopoietic regeneration (Cavaletti and Marmiroli 2010; Lucas et al. 2013). In particular, since sympathetic nerve fibers in BM are closely associated with HSC function, neuroprotection is important for the rescue of chemotherapy-induced HSC dysfunction (Aloe et al. 2000; Lucas et al. 2013). In our previous studies, NPY was shown to promote neuroprotection, resulting in mitigation of cisplatin-induced HSC dysfunction by protecting nerve injury in BM (Park, Jin et al. 2015; Park, Min et al. 2015). Importantly, NPY-mediated protective effect did not affect the anticancer efficacy of cisplatin therapy in an ovarian cancer mouse model (Park et al. 2017). Here we first characterized specific sequences of NPY for the improvement of cisplatin-induced bone marrow damage. NPY (1–15) and NPY (6–20), derived from the full-length NPY, significantly restored HSC impairment in cisplatin-treated mice. We also observed that cisplatin-induced sensory neuropathy was prevented by these peptides, as they reduced cisplatin-induced apoptosis of Th^+^ fibers in bone marrow microenvironment. *In vitr*o experiments, examining PC12 cell-differentiation into neurons, showed that the CM derived from NPY (1–15)- or NPY (6–20)-treated macrophages induced neural differentiation of PC12 cells by elevating TGF-β secretion from the macrophages. These results suggested that the two new peptides may be potent as therapeutic agents for chemotherapy-induced bone marrow abnormalities.

Notably, NPY (6–20) showed more protective effect against cisplatin-caused bone marrow damage than the full-length NPY, indicating that this peptide contains the most effective sequence for rescuing the chemotherapy-induced bone marrow dysfunction. We recently identified NPY (6–20) for HSC mobilization that results in an amelioration of ovariectomy-induced bone loss by reducing osteoclast deposition on the bone surfaces. The peptide induced HSC mobilization and ameliorated bone loss in ovariectomized mice more effectively compared to the effect of full-length NPY (Park et al. 2017). Taken together, our findings demonstrate that NPY (6–20) plays a bimodal role on the regulation of HSC function in BM and could be used for the treatment of both osteoporosis and chemotherapy-related bone marrow failure.

During the past decade, peptides have gained increased attention as therapeutic tools in various diseases (Vlieghe et al. 2010; Fosgerau and Hoffmann 2015), because the peptides used in drugs are easy and inexpensive to produce, and the ones synthesized from natural peptides, already present physiologically, have high specificity and low toxicity. Despite these attractive features of peptides, poor physico-chemical stability and a short circulating half-life in the plasma limit their use as convenient therapeutics (Vlieghe et al. 2010; Otvos and Wade 2014; Fosgerau and Hoffmann 2015). However, recent studies suggest that these weaknesses can be overcome by physical and chemical modifications of the peptides influencing their spatial structure (Otvos and Wade 2014; Fosgerau and Hoffmann 2015). NPY, which is naturally produced in the human body, also has therapeutic benefits including high potency, activity and speciﬁcity for a target receptor, lack of toxic metabolites, and lower accumulation in tissues (Robertson et al. 2011). Therefore, NPY-derived peptides would warrant these advantages as well. Since the half-life of NPY in peripheral plasma is known to be approximately 20–28 min (Potter 1987), the NPY-derived peptides may also be expected to have a short half-life. The structure of NPY is already known; some researchers have already attempted to modify NPY sequence to improve its properties (Allen 1990; Beck-Sickinger et al. 1994). We consider that further research, focused on the modification of these peptides, may enhance their potential as clinical therapeutic agents against other chemotherapy-induced side effects.
